# Enhancing the Fire Resistance of Ablative Materials: Role of the Polymeric Matrix and Silicon Carbide Reinforcement

**DOI:** 10.3390/polym16172454

**Published:** 2024-08-29

**Authors:** Juana Abenojar, Sara López de Armentia, Miguel Angel Martínez

**Affiliations:** 1 Materials Science and Engineering Department, Universidad Carlos III de Madrid, 28911 Leganés, Spain; mamc@ing.uc3m.es; 2 Mechanical Engineering Department, Universidad Pontificia Comillas, 28015 Madrid, Spain; sara.lopez@comillas.edu; 3 Institute for Research in Technology, Universidad Pontificia Comillas, 28015 Madrid, Spain

**Keywords:** ablative material, wear, impact, silicon carbide, polypropylene, polyester

## Abstract

The primary characteristic of ablative materials is their fire resistance. This study explored the development of cost-effective ablative materials formed into application-specific shapes by using a polymer matrix reinforced with ceramic powder. A thermoplastic (polypropylene; PP) and a thermoset (polyester; UPE) matrix were used to manufacture ablative materials with 50 wt% silicon carbide (SiC) particles. The reference composites (50 wt% SiC) were compared to those with 1 and 3 wt% short glass fibers (0.5 mm length) and to composites using a 1 and 3 wt% glass fiber mesh. Fire resistance was tested using a butane flame (900 °C) and by measuring the transmitted heat with a thermocouple. Results showed that the type of polymer matrix (PP or UPE) did not influence fire resistance. Composites with short glass fibers had a fire-resistance time of 100 s, while those with glass fiber mesh tripled this resistance time. The novelty of this work lies in the exploration of a specific type of material with unique percentages of SiC not previously studied. The aim is to develop a low-cost coating for industrial warehouses that has improved fire-protective properties, maintains lower temperatures, and enhances the wear and impact resistance.

## 1. Introduction

Ablative materials absorb thermal energy through endothermic phase transitions, causing irreversible structural and chemical changes while absorbing heat. Heat shields balance convective heat with surface radiation, phase transitions, and chemical reactions, and they also block the incoming heat from hot gases from degradation, maintaining the surface temperature and increasing the surface recession rate with higher heat fluxes [1,2].

A thermal protection system (TPS) shields aerodynamic surfaces, protecting spacecraft and missiles from extreme heat by either absorbing or dissipating heat sacrificially [3]. Near space has gained importance in aerospace, with hypersonic aircraft experiencing high Mach numbers and extended flight times leading to heat accumulation, particularly at the noses and leading edges. Current research focuses on TPS materials for hypersonic aircraft in severe aero-thermodynamic environments [4].

Phenolic resin (PR) composites, particularly carbon/phenolic (C/PR) composites, are widely used in aerospace as TPS materials due to their excellent ablation resistance, which make them suitable for high enthalpy and high heat-flux environments [5]. However, their high density and increasing thermal conductivity at elevated temperatures reduce their insulation capacity. Phenolic-impregnated carbon ablator (PICA), with its low density and efficient heat dissipation, is a leading TPS material due to its porosity [6,7,8]. Despite its excellent anti-ablation properties, PICA undergoes significant surface recession during extended flights. PICA materials show better thermal insulation than traditional C/PR composites [9,10].

Carbon-fiber-reinforced composites with a phenolic matrix (CF/PR) have been used in rocket nozzle components over the last 20 years due to their thermal stability and resistance to high-temperature ablation and oxidation [11]. However, there is ongoing interest in improving the ablation resistance of CF/PR composites to meet the demand for lighter and thinner ablative materials. Studies have incorporated carbon-based materials like carbon nanofibers [12], carbon nanotubes (CNTs) [13], CNTs together with carbon black [14], and graphene oxide (GO) [15,16] into the phenolic matrix or as anti-ablative coatings. In addition, ceramics such as zirconia [17] or nanoboron carbide (NBC) [4] can be attached to these carbon-based materials, forming composite materials with NBC/PR as a matrix and carbon fiber as reinforcement. Thus, the boron oxide formed from boron carbide conversion greatly improves dimensional stability, maintaining structural integrity after combustion [4].

As a more specific example, needle-punched carbon fiber/phenolic resin (NCF-PR) composites, created via vacuum impregnation using NCF as the matrix and PR aerogels as fillers, are effective ablative materials. The aerogels’ high porosity and mesoporosity yield low densities (0.270–0.370 g/cm^3^) and low thermal conductivities (0.093–0.230 W/mK) at room temperature. These lightweight composites are ideal for thermal insulation and protection in energy-saving and aerospace applications [3]. Molecular dynamics simulations reveal that even a small concentration of GO within the matrix promotes the growth of graphitized crystals in carbonized PR [16], and this is associated with enhanced thermal resistance.

On the other hand, phenolic composites with quartz or silica fibers [18,19] have good heat insulation properties but a low melting point, which limits their performance at high working temperatures and results in insufficient ablation resistance. Ceramic matrix composites such as C/C, C/SiC [20], and SiC/SiC [21] have been developed as a coating for use in ultra-high-temperature environments, such as leading edges, but they tend to have relatively high densities.

Simulation methods are crucial for evaluating new materials’ thermal protection efficiently and cost-effectively. High-fidelity FEM solvers enhance accuracy in simulating thermal and ablation behaviors but need better integration of processing parameters and material performance [22]. Li et al. developed a multifunctional ablative material to improve the insulation and ablation resistance and to reduce weight. Their model includes manufacturing parameters, aiding in the thermal protection design for hypersonic vehicles [23]. Additionally, Li et al. also added local thermal non-equilibrium and thermal radiation mechanisms, showing the impact of pore size and porosity on current ablative materials [24].

Ablative materials can also be used in fire protection and as fire-spread retardants, and nanotechnology has been explored in fire safety [25]. Fire risks can be reduced by integrating fire retardants (FR) into polymer matrices or by applying FR coatings [26,27]. FRs cause charring, forming a protective barrier that enhances the FR effects [26,28,29]. Additives like graphene oxide can be functionalized to create nanocomposite FRs with a thermoplastic matrix [30]. Hybridizing FRs through covalent bonds with inorganic nanoparticles offers diverse strategies for FRs [31].

Given the complexity, cost, and environmental impact of existing materials, there is a need for low-cost materials. Although these materials may not perform as well, they can still be useful for less demanding industrial applications, such as coatings in industrial warehouses to slow down flames or to allow evacuation. According to Portocarrero et al. [32], low-cost ablative materials can also be used in rocket nozzles. These ablative composites consist of a polyester resin matrix reinforced with 60–70% by weight of particulate materials and 5% by weight of short fiberglass. The particulates used are finely ground ceramic residues and aluminum dross (<75 μm), which are both industrial byproducts.

Building on the context mentioned, this study aims to investigate the fire behavior of a composite material made from polyester resin (UPE) or polypropylene (PP) with 50 wt% of 43 μm SiC particles supplemented with 1–3 wt% short glass fibers (GFs). Additionally, the influence of adding a GF mesh was examined in the best-performing polymer. The novelty of this work lies in its unique objective, since no previous literature has explored this specific type of material, especially with these SiC percentages. In addition, the final application (coatings for industrial warehouses) requires examining properties such as wear and impact resistance. The goal is to develop a low-cost coating that can protect structures from fire, maintain lower temperatures for longer periods, and improve the wear and impact resistance.

## 2. Materials and Methods

### 2.1. Materials

Polypropylene (PP) is a recyclable, non-charring thermoplastic polymer that is used as the matrix for the composite material. PP was provided in pellet form, with a diameter of 3 mm and a density of 0.92 g/cm^3^, which was supplied by Repsol (Madrid, Spain). Its key properties include stiffness, transparency, brightness, surface hardness, chemical resistance, temperature resistance, gas barrier properties, mechanical strength, low UV degradation, and low surface energy.

Unsaturated polyester resin (UPE), specifically Ferpol 3501, was used as the matrix for the manufactured composites. Supplied by Feroca S.A. (Madrid, Spain), it has low viscosity (250 mPa·s), a density of 1.2 g/cm^3^, and low reactivity. For curing, an accelerator (CH-8) and a catalyst (F-11) are needed in a ratio of 100:2:2.5, both provided by Feroca S.A. (Madrid, Spain). This charring thermoset polymer is known for its high strength and toughness, abrasion resistance, heat resistance, low creep at high temperatures, chemical resistance, dimensional stability, ease of working, and cost-effectiveness.

**Figure 1:**
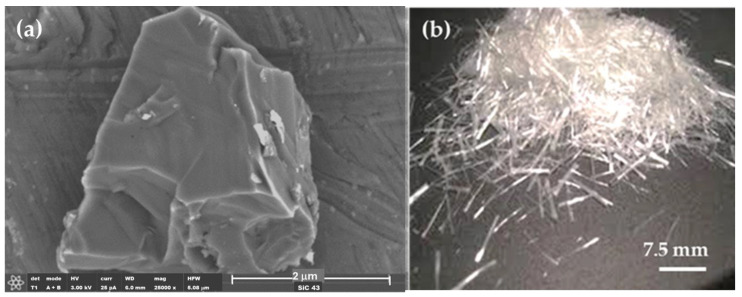
(**a**) Micrograph of SiC. (**b**) Macrograph of GFs taken with a mobile phone camera.

Silicon carbide (SiC) particles, sized at 43 μm with a polygonal shape as shown in Figure 1a, were provided by Carburos Navarro (Cuenca, Spain) and have a density of 3.21 g/cm^3^.

Glass fibers (GFs) were used in amounts of 1 and 3 wt%. These short fibers, each 6 mm long and with a diameter of 2 ± 0.5 mm, have a density of 2.38 g/cm^3^ (Figure 1b). The mesh density is 1.69 g/cm^3^, and the mesh size is 3.50 mm. The GF and mesh densities were calculated by Archimedes’ principle, and both were supplied by Feroca S.A. (Madrid, Spain).

### 2.2. Sample Preparation

Polypropylene (PP) composites were manufactured by melting and blending the polymer with silicon carbide particles using a Haake Rheomix 252P (Waltham, MA, USA) at 190 °C and 50 rpm for 6 min. The resulting mixture was then processed to be fabricated into a 3 mm thick plate using a hot plate press (FontijnePresses TPB374, Barendrecht, The Netherlands) at 190 °C and 5.5 MPa for 20 min.

UPE composites were prepared by mechanically mixing the components for 15 min at 180 rpm in a mechanical mixer (Supertest Air, VMI Rayneri, Montaigu Vendée, France). The mixture was then poured into silicone molds for curing, with different sample sizes molded depending on the intended tests. Mesh sheets were manually placed into the silicone molds and cut according to the test requirements. Samples were cured at room temperature (21–22 °C) for 24 h before testing, and all materials were tested one week after fabrication. The nomenclature of the tested samples is provided in Table 1.

### 2.3. Fire Test

**Figure 2:**
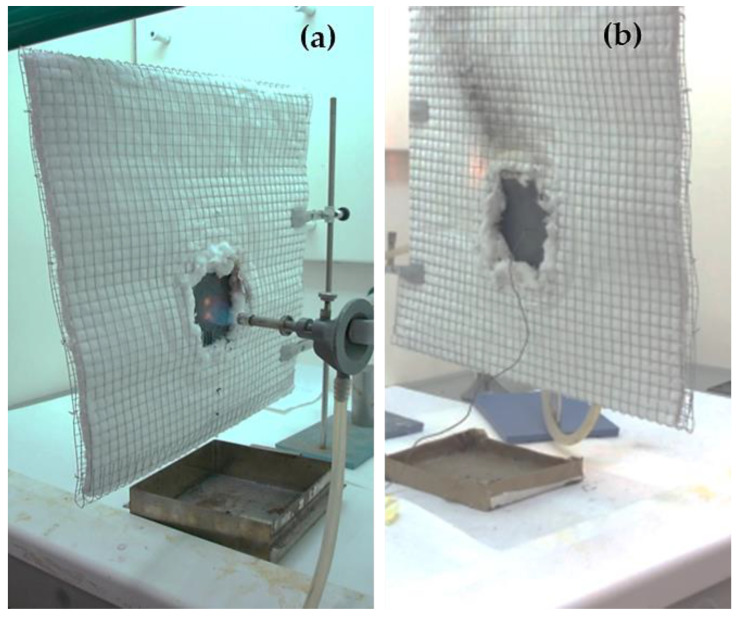
Fire test setup: (**a**) in front of the sample, (**b**) behind the sample.

Three sheets of 100 mm × 100 mm with a thickness of 3.5 mm were fabricated for each composite to conduct fire tests. These tests involved exposing the samples to a flame at 900 °C (measured with a thermocouple) from a Bunsen burner fueled by butane gas. The reducing zone of the flame was in contact with the material during the test (Figure 2a). The temperature on the side opposite to the flame was measured with a thermocouple (Figure 2b). The test was continued until the flame passed to the other side of the sample, and each composition was tested three times.

### 2.4. Wear Test

Dry wear tests were conducted at room temperature using a pin-on-disk tribometer (Microtest, Madrid, Spain) with a 6 mm diameter alumina ball as the pin. The test conditions were set to 120 rpm, with an applied load of 15 N, relative humidity below 30%, and a friction radius of 8 mm. The sliding distance was 1000 m. For each composition, three samples, each measuring 25 mm on each side and with a thickness of 1.1 mm, were used.

The wear and coefficient of friction were calculated according to the ASTM G99 05 (2010) standard test [33]. Wear was assessed by measuring volume loss using Archard’s equation (Equation (1)). Volume loss was determined relative to the wear track (Equation (2)), with the wear track radius (R) and wear track width (d) measured using a DSX500 opto-digital microscope (OM) provided by Olympus Corporation (Tokyo, Japan) and (r) representing the pin end radius. It was assumed that there was no significant wear on the pin, and the wear debris was intentionally left on the track, as it plays a crucial role in fretting wear models [34].

(1) $$W=\frac{Volume\;loss}{Load\times Sliding\;distance}\left(\frac{{mm}^{3}}{N\cdot m}\right)$$

(2) Volume loss (mm3)=2πRr2sin−1d2r−d44r2−d21/2

Later, wear tracks were analyzed by scanning electron microscopy (SEM) using the Philips X-30 model (Philips Electronic Instruments, Mahwah, NJ, USA) to determine the mechanism of wear [35].

### 2.5. Impact Test

Charpy impact tests were carried out in the pendulum impact-testing machine CEAST 9050 Instron^®^ (Barcelona, Spain), according to the ASTM E23 standard [36]. The size of the samples was 10 mm × 1.1 mm × 220 mm, and five samples of each composition were tested. The absorbed energy in the Charpy impact test (E) was obtained by Equation (3) and is directly calculated by the machine’s program; the used parameters are the mass of hammer (W), gravitation acceleration (g = 9.80655 m/s^2^), length (R), angle at the end the swing (β), angle of fall (α), and energy loss (L). The resilience was obtained through Equation (4) using the absorbed energy and sample area.

(3) $$Absorbed\;Energy\left(kJ\right)=W\left(kg\right)\times g\left(\frac{m}{s^{2}}\right)\times R\left(m\right)\times \left(cos\;\beta -cos\;\alpha \right)-L(J)$$

(4) $$Resilience=\frac{Absorbed\;Energy\left(kJ\right)}{So\left(m^{2}\right)}$$

### 2.6. Differential Scanning Calorimetric (DSC) Technique

DSC analysis (DSC 822e, Mettler Toledo GmbH, Greifensee, Switzerland) was performed on the polymer sheets to identify possible changes in the thermal properties of PP composites with the addition of SiC and/or GF. The DSC was performed at a heating rate of 20 °C/min in a temperature range of 0 °C to 200 °C. Aluminum crucibles of 40 µL were used and filled with 9–10 mg of PP or PP composites. Moreover, nitrogen, as a purge gas, was fed at a rate of 50 mL/min. STARe Evaluation V12.10 software provided by Mettler Toledo was employed to calculate the degree of crystallinity and the melting temperature. The used melting enthalpy of the 100% crystalline PP was 207 J/g [37].

## 3. Results 

### 3.1. Fire Test

#### 3.1.1. Polypropylene

**Figure 3:**
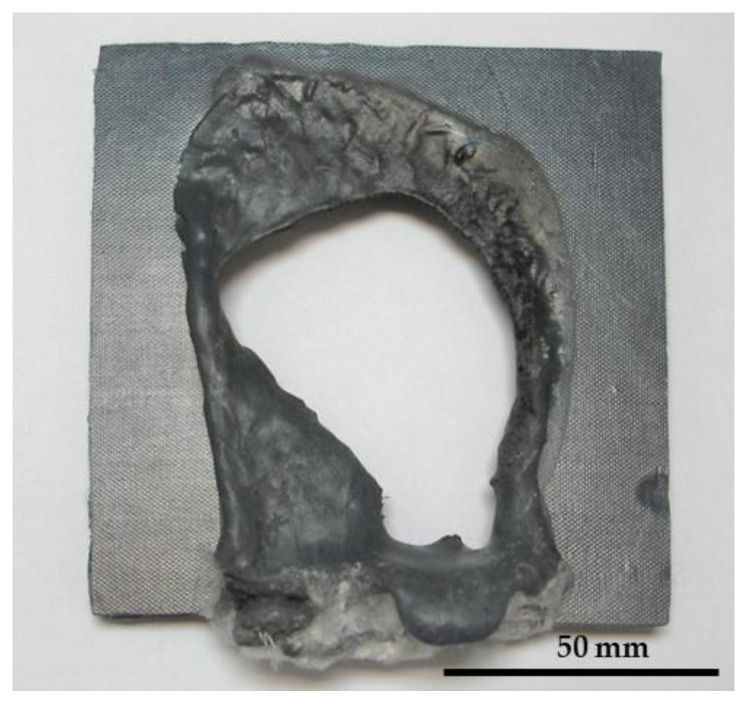
PP + 50SiC + 3GF sample after the fire test.

PP matrix composites did not fracture when exposed to flame and showed a certain fire resistance. However, once the melting point of the matrix was reached, the composite started to melt (Figure 3). The maximum time (Table 2) before the flame penetrated the composite was 105 s, with the thermocouple reaching 185 °C for the PP + 50SiC + 3GF composite. Additionally, PP does not char; instead, it drips as it melts.

#### 3.1.2. Polyester

**Figure 4:**
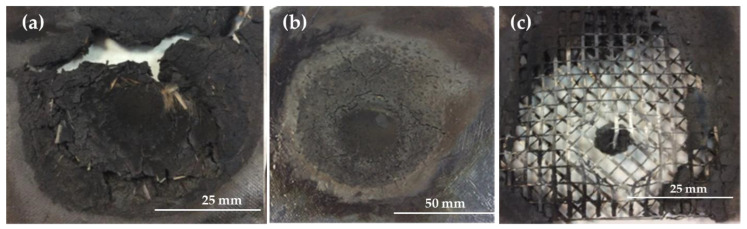
UPE composites after the fire test (**a**) UPE + 50SiC + 3GF, (**b**) UPE + 50SiC + mesh at 200 s and (**c**) UPE + 50SiC + mesh at the end of test.

**Figure 5:**
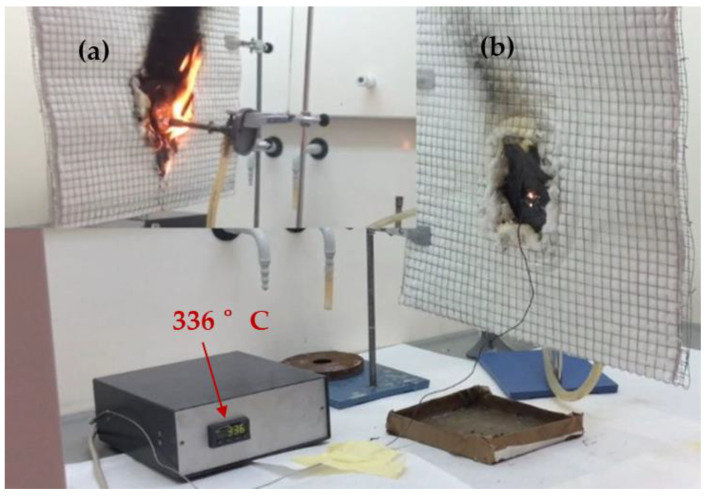
Fire test setup for UPE + 50SiC + mesh: (**a**) side where the fire hit, (**b**) opposite side to the fire, connected to a thermocouple.

The use of a polyester matrix prevented melting because it is a charring thermoset polymer (Figure 4a), although the fire tests were still relatively short (Table 3). However, when the short fibers were replaced with glass mesh (Figure 5) in the same proportions, the temperature measured by the thermocouple reached 336 °C (Figure 4b) before the flame passed, and the time increased to 430 s (Table 3). At around 200 s, the composite with the UPE + 50SiC + mesh did not show any visible cracks (Figure 4b) and retained its shape until the end of the test; this was due to the mesh (Figure 4c).

### 3.2. Wear Tests

**Figure 6:**
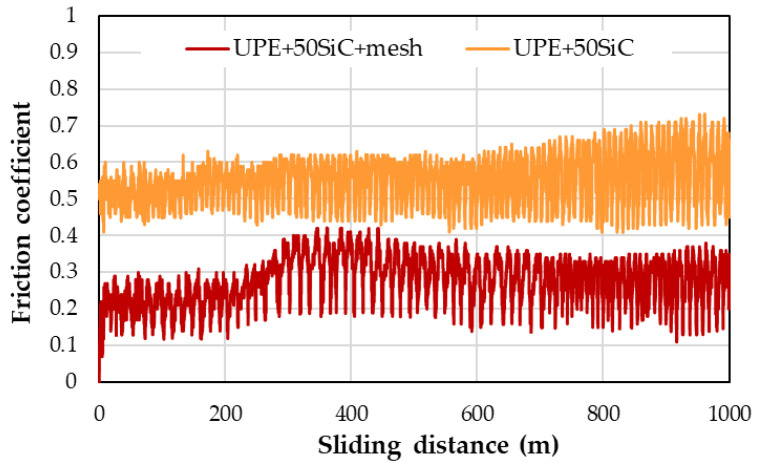
Examples of friction coefficient curves for UPE + 50SiC and UPE + 50SiC + mesh.

Figure 6 shows the typical curves for these materials. For UPE + 50SiC, the FC curve initially increased over the first few meters and then reached a stable value, with fluctuations that slightly increased until the end of the test. When the mesh was added, the FC was lower, and a peak around 350 mm appeared; then, the FC decreased gradually until the end (Figure 6).

**Figure 7:**
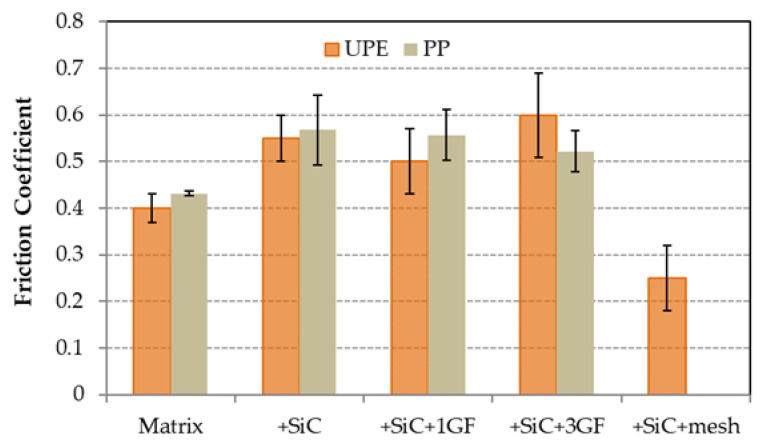
Friction coefficients for PP and UPE matrices and their composites.

The friction coefficient (FC) for the studied matrices and composites is shown, with each value representing the average of three samples (Figure 7). The inclusion of SiC, due to its abrasive nature, increased the FC for both matrices. While short glass fibers did not have a significant impact on the FC, their random distribution appears to contribute to a slight increase. The addition of mesh notably reduced the FC in the UPE composite by approximately 40%, making it the composite with the lowest FC observed in this study.

**Figure 8:**
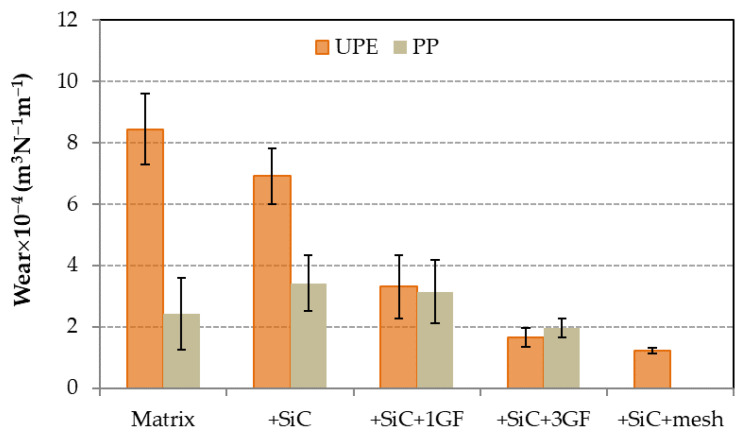
Wear of PP and UPE matrices and their composites.

The wear of the matrices was quite different, as it depended on their rigidity and relative density. The wear of PP was 71% less than that of UPE (Figure 8), indicating different wear mechanisms for each matrix. The wear of the UPE composites progressively decreased with the addition of SiC and GF, reaching the lowest value (−85%) for UPE + 50SiC + mesh. Conversely, the wear of PP composites increased by 38% with the addition of SiC, likely due to increased rigidity. However, for the composite with 3GF, the wear decreased slightly.

**Figure 9:**
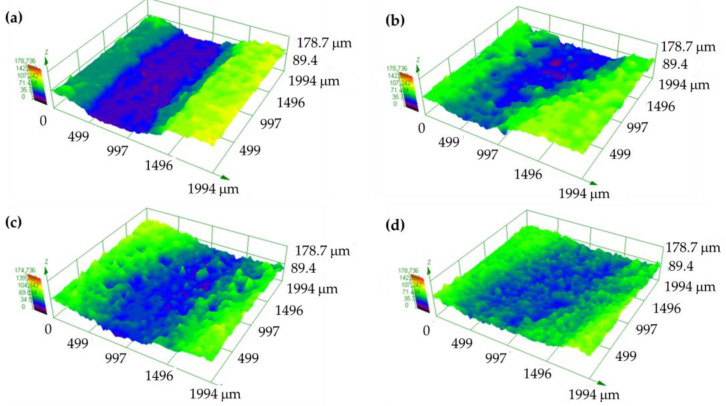
3D panoramic wear tracks by OM: (**a**) PP, (**b**) PP + 50SiC, (**c**) PP + 50SiC + 1GF, and (**d**) PP + 50SiC + 3GF.

Figure 9 depicts the wear tracks in 3D images by OM. The PP track (Figure 9a) appeared clearer than the composite tracks, with a higher depth for PP + SiC (Figure 9b). However, the composite tracks showed more accumulation at the edges when GF was added (Figure 9c,d). The track width values for PP, PP + SiC, PP + SiC + 1GF, and PP + SiC + 3GF were 1308 ± 18 μm, 2157 ± 134 μm, 1816 ± 83 μm, and 1286 ± 103 μm, respectively. The depth also changed, although these changes were smaller, measuring around 80 ± 15 μm for PP and PP + SiC and around 75 ± 19 μm for PP + SiC + 1GF and PP + SiC + 3GF.

**Figure 10:**
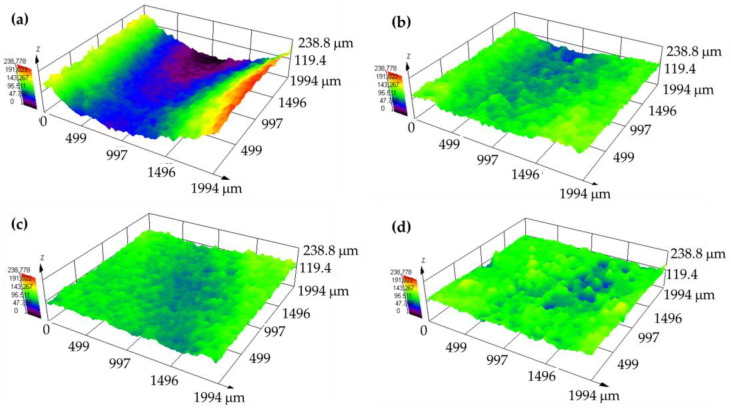
3D panoramic wear tracks by OM: (**a**) UPE, (**b**) UPE + 50SiC, (**c**) UPE + 50SiC + 3GF, and (**d**) UPE + 50SiC + mesh.

The wear tracks for UPE and its composites showed distinct differences. For UPE, the wear tracks were deeper and lacked edge accumulation (Figure 10), whereas the wear tracks in the composites were flatter, particularly as the glass fiber (GF) content increased (Figure 10b–d). In the composites, accumulation was observed at the edges and along the tracks, which affected the wear behavior.

The track width values for UPE, UPE + SiC, UPE + SiC + 1GF, UPE + SiC + 3GF and UPE + SiC + mesh were 1896 ± 103 μm, 1677 ± 122 μm, 1570 ± 38 μm, 1548 ± 71 μm, and 1206 ± 51 μm, respectively. The depth also changed, being 161 ± 23 μm 52 ± 10 μm, 40 ± 10 μm, 39 ± 11 μm, and 23 ± 2 μm for UPE, UPE + SiC, UPE + SiC + 1GF, UPE + SiC + 3GF, and UPE + SiC + mesh, respectively.

**Figure 11:**
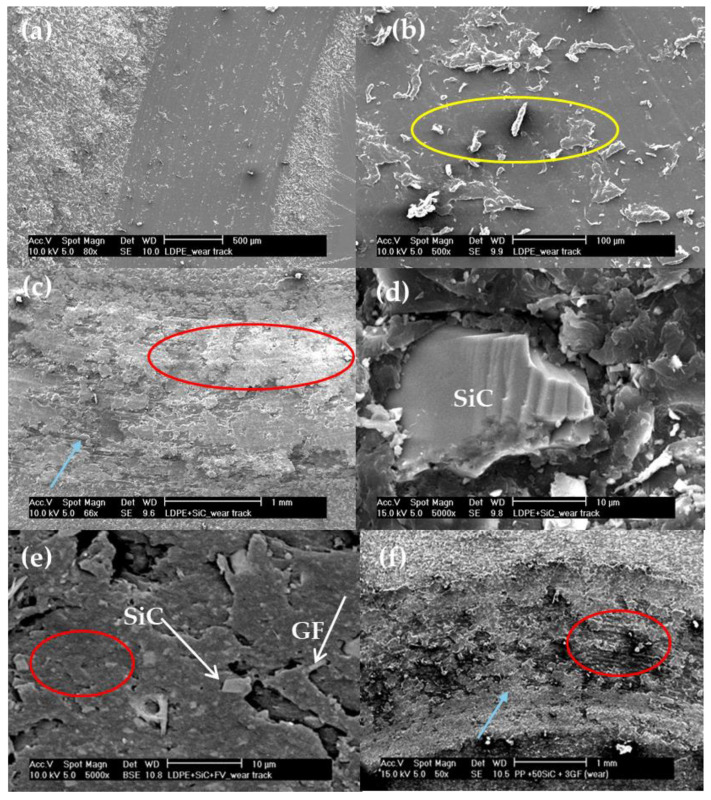
Micrograph of wear tracks: (**a**) PP track; (**b**) detail of the PP track: debris powder (yellow circle); (**c**) PP + SiC track, accumulation area (red circle) and abrasion line (blue arrow), (**d**) PP + SiC track, showing the detail of the SiC particle on the track; (**e**) PP + SiC + 1GF track, showing the SiC particle and GF in the accumulation track (red circle); and (**f**) PP + SiC + 3GF track, showing the accumulation area (red circle) and abrasion line (blue arrow).

At low magnification, the wear track of PP appeared to be clean and quite uniform (Figure 11a). When examined at higher magnification, some debris powder was visible (Figure 11b), but there were no signs of accumulation or agglomerates. However, with the addition of SiC, accumulation or agglomerates formed on the track (red cicle), along with visible abrasion lines (blue arrow) (Figure 11c). Within these agglomerates, SiC particles could be found, which exhibited abrasive or fatigue lines on their surfaces (Figure 11d). GF particles were also visible within the accumulated powder on the track (Figure 11e,f), along with areas showing abrasion lines (Figure 11f).

**Figure 12:**
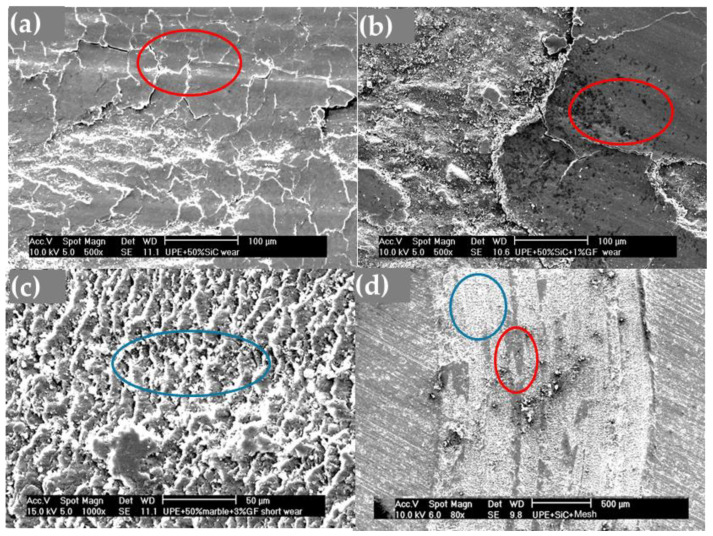
Micrographs of wear tracks: (**a**) UPE + 50SiC, (**b**) UPE + 50SiC + 1GF, (**c**) UPE + 50SiC + 3GF, and (**d**) UPE + 50SiC + mesh. (Red circles mark areas of agglomerates or deposits, and blue circles mark areas of abrasion and fatigue).

The micrographs of the wear tracks of the UPE composites can be observed in Figure 12. In UPE + SiC (Figure 12a), zones of accumulation, deposits, or agglomerates were clearly visible (red circles), while in Figure 12b, corresponding to UPE + SiC + 1GF, both abrasive zones (blue circle) and deposits (red circles) could be found. In Figure 12c (UPE + SiC + 3GF), abrasive or fatigue zones (blue circle) predominated. For UPE + SiC + mesh (Figure 12d), abrasive zones were more prevalent at the edge of the track (blue circle), and in the central part, deposits and some debris powder were found, which appeared to coincide with a mesh fiber (red circle).

### 3.3. Impact Test

**Figure 13:**
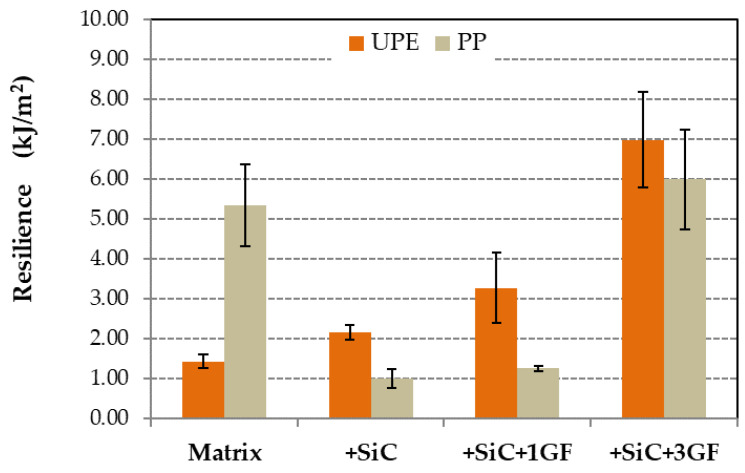
Resilience values obtained from impact tests for both matrices and their composites.

Figure 13 shows the resilience values calculated from the impact test according to Equation (4). PP composites had less resilience than the PP matrix; only when 3% GF was added was the resilience was higher than in the pure matrix. However, for the UPE matrix, the resilience increased for all the composites gradually. The impact test showed high energy absorption for UPE + 50SiC + mesh, showing 74 kJ/m^2^ compared with 7 kJ/m^2^ for the composite with 3% short GFs. These values were considerably higher than those of UPE + 50SiC or UPE, being 2.15 kJ/m^2^ and 1.42 kJ/m^2^, respectively. Error bars were affected by the placement of the fibers in the sample. The UPE + 50SiC + mesh did not break, with the parts remaining attached.

**Figure 14:**
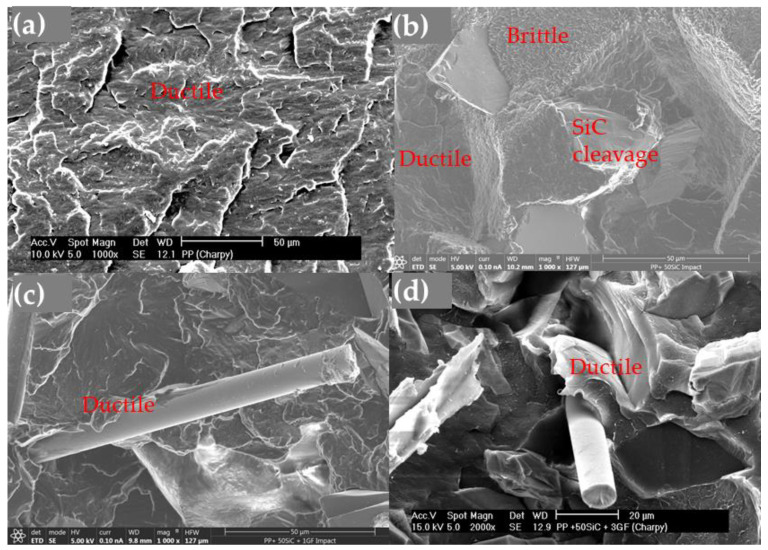
Micrographs of the impact test fracture surface: (**a**) PP fractography; (**b**) PP + SiC fractography with ductile and brittle areas and SiC cleavage; (**c**) PP + SiC + 1GF fractography, showing the detail of the GF in ductile area; (**d**) PP + SiC + 3GF track, showing the detail of the GF in ductile area.

The fracture surfaces of PP exhibited characteristics typical of ductile failure (Figure 14a), showing a deformed aspect. When SiC was added, the fracture surface changed, revealing SiC particles with cleavage and areas with a brittle appearance (Figure 14b). The glass fibers (GFs) in the composites needed to be pulled out and broken, with the PP matrix stretching around the GF (Figure 14c,d), thereby increasing the resilience.

**Figure 15:**
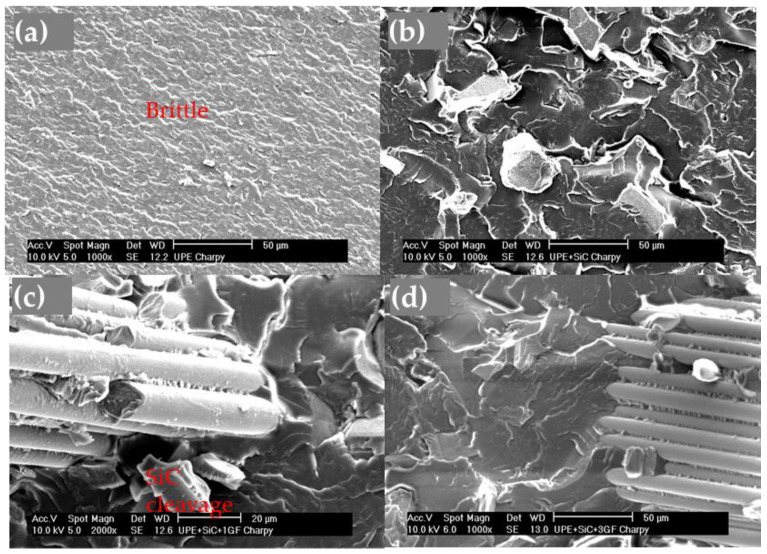
Micrographs of the impact test fracture surface: (**a**) UPE fractography; (**b**) UPE + SiC fractography, (**c**) UPE + SiC + 1GF fractography, showing the SiC cleavage and GFs; and (**d**) UPE + SiC + 3GF fractography, showing the accumulation of GFs.

After the impact test, the UPE surface presented a smooth and glossy aspect, as shown in Figure 15a, which corresponded to a brittle fracture. The fracture surface changed when SiC was added. The particles absorbed energy from the impact, and the SiC particles broke by cleavage (Figure 15b), which provided a low deformation compared with UPE. In composites with GF, the fibers need to be pulled out and broken, increasing resilience and toughness (Figure 15c,d).

## 4. Discussion

### 4.1. Fire Resistance

#### 4.1.1. Polypropylene

Polypropylene (PP) is a non-charring thermoplastic polymer with a melting temperature of 175 °C (Table 4), as measured by differential scanning calorimetry (DSC) at a rate of 20 °C/min. The melting temperature (Table 4) decreases to 169 °C with the addition of silicon carbide (SiC) and further decreases to 160 °C with the addition of 3% glass fiber (GF). Despite this reduction in melting temperature, the crystallinity of PP increases from 31% to 37% with the addition of SiC, and it remains close to that of pure PP (around 30%) when GF is added (Table 4). SiC particles can be considered nucleating agents [38], while GF does not have this effect. The decreased melting temperature may be due to weaker bonds formed from the addition of more fiber and polymer chains of lower molecular weight.

This reduction in melting temperature did not impact the fire test performance. The composite PP + 50% SiC + 3% GF exhibited improved fire resistance, providing more time before melting and dripping. SiC particles absorb heat without undergoing a chemical reaction. However, evenly distributed SiO_2_ (silica) can develop a shell-like structure when heated to ignition [39], which is rich in inorganic compounds and exhibits excellent barrier properties. This silica layer functions as an insulating membrane, reducing heat and oxygen transport and lowering smoke generation. GF contains silica and can contribute to forming this barrier, providing a slight improvement in the fire resistance of PP [40].

Throughout the test, no smoke was detected. Since the material is composed of only 47% polymer, the potential for smoke emission is significantly reduced compared with a material made entirely of polymer.

#### 4.1.2. Polyester Resin

The decomposition of polyester resin begins at approximately 350 °C. Beyond this temperature, the resin undergoes rapid decomposition through an endothermic process, which is primarily driven by random chain scission of the main polymer chain. Significant weight loss occurs at around 480 °C, with the polyester retaining less than 5% of its initial mass as char [41]. When GFs were added, the decomposition process occurred at higher temperatures and the residual mass at 500 °C increased due to the thermal stability of GF at temperatures below 1000 °C [42].

According to the literature, polyester resins are typically modified with phosphate-based fire retardants such as ammonium polyphosphate, silane-coated ammonium polyphosphate, and melamine pyrophosphate. These fire retardants provide a special composition of char that acts as a protective layer. This protective char contains NH_3_, metaphosphoric acid, and SiO_2_ particles. NH_3_ can reduce the concentration of combustible gases in the gas phase, while the metaphosphoric acid facilitates the formation of char layers in the condensed phase. Additionally, SiO_3_ particles can be formed in the char residues from the reaction between silane and oxygen. Thus, the combination of protective char layers and the reduced concentration of combustible gases significantly improves fire safety properties [43,44]. In the case of UPE, slight smoke emission was observed at the conclusion of the test. However, this emission is still lower than what would be expected if the material were composed of 100% polymer.

The GF used in this work, whether as short fibers or mesh, provides silica, which delays the degradation of UPE. In the case of the mesh, it also offers support for the char, thereby improving the mechanical stability of the material.

### 4.2. Wear Behaviour

The friction coefficient (FC) versus sliding distance curves exhibit two distinct phases. Initially, the FC increases rapidly, reaching a peak value typically associated with track formation, primarily through abrasive wear mechanisms [45]. For most materials, this phase occurs after approximately 100 m of sliding, though it varies depending on the abrasive mechanism. Interestingly, for PP and UPE composites, the FC stabilizes after a few meters without displaying a clear maximum peak (Figure 6).

In the subsequent phase, the FC remains relatively constant, albeit with slight variations that are likely due to alternating adhesion and abrasion mechanisms. The average FC value during this phase is often reported as the steady-state friction coefficient. However, stabilization may not be complete due to the ongoing influence of abrasive particles in the track, known as the third-body effect, which intensifies abrasion [46]. Figure 6 illustrates that the average FC value remains fairly constant throughout the wear tests on UPE and PP. Fluctuations arise from the formation and growth of wedges, which are caused by material deformation and the retention by loose particles that accumulate into debris dust [47]. This debris persists throughout the test, forming wedge-like agglomerates by the end.

The wear mechanism in this scenario is primarily abrasive–adhesive, which can either sustain or diminish the FC. This behavior is evident in UPE and PP composites, where the FC remains similar with the addition of SiC but showing minor variations with the addition of GF. Notably, the FC decreases significantly with the addition of mesh, likely due to its parallel positioning to the sliding track, which facilitates easier movement (Figure 7).

However, variations are noted within each measurement, possibly due to wedge-shaped agglomerates. These agglomerates can become detached during wear because they lack proper anchoring to the matrix.

Optical microscopy (OM) reveals the presence of accumulations on the wear tracks, indicating an adhesive mechanism. This observation aligns with a sequence where abrasive wear is followed by adhesive wear, which is characteristic of polymers and composites [48].

The wear resistance of both matrices differs (Figure 8). PP is a ductile polymer, whereas UPE is brittle. As a result, PP can undergo deformation, creating a wear track that is narrower and deeper (Figure 9a) compared with UPE (Figure 10a). This indicates that PP exhibits less wear than UPE under identical test conditions. Furthermore, the wear mechanisms also differ: plastic deformation for PP and abrasive wear for UPE, as illustrated in the SEM micrographs (Figure 11a and Figure 12a).

On the other hand, wear in composites with SiC appears to be abrasive–adhesive. The angular and pointed shape of SiC microparticles (Figure 1a) logically leads to increased wear in composites due to the favorability of abrasive wear (Figure 8), as observed in PP + SiC. The potential presence of particle agglomerates within the composite material, which upon detachment create more free volume, may contribute to increased wear. This phenomenon involves material pull-out and the formation of wedge-like agglomerates, resulting in wider wear tracks for PP + 50SiC and PP + 50SiC + 1GF (Figure 9b,c). Nevertheless, the overall mechanism remains abrasive–adhesive. Glass fibers (GFs) introduce adhesive wear, especially when oriented in the sliding direction, facilitating the adhesion of debris powder. In such cases, narrower wear tracks are evident, as seen in PP + 50SiC + 3GF (Figure 9d).

The behavior of UPE composites differs, with increased wear resistance resulting in less wear compared with pure UPE (Figure 8). Even UPE + 50SiC exhibits a narrower and less deep wear track than UPE, primarily due to an adhesive mechanism (Figure 10b), which is particularly pronounced in edge tracks where debris powder accumulates. This third body could protect the matrix and, consequently, wear decreases [49]. Moreover, wear resistance improves with the addition of GFs, as the adhesive mechanism predominates in UPE + 50SiC + 3GF and UPE + 50SiC + mesh. In these latter cases, wear tracks are nearly covered, resulting in shallow wear tracks (Figure 10c,d).

Typically, agglomerates resulting from adhesive wear tend to accumulate more on the edges of the track. However, they are also distributed throughout the track, causing fluctuations in the friction coefficient (Figure 6). The center of the track experiences more abrasive wear, which results in its non-flat nature and higher depth.

The wear behavior can be comprehended through various mechanisms, in which the interaction between particles and the matrix is crucial. Typically, wear tracks display abrasive–adhesive characteristics, as illustrated in Figure 11 and Figure 12. Abrasion lines (highlighted by blue circles and arrows) are visible in Figure 11f and Figure 12c,d while wedge-shaped agglomerates (indicated by red circles) appear in Figure 11c,e,f, and Figure 12a,b,d. The fatigue mechanism is also present alongside the abrasion mechanism (blue circles) in Figure 12c. Abrasion lines are particularly pronounced in composites containing 50SiC, leading to a high FC (Figure 7).

Fatigue wear results from cyclic mechanical stresses, which promote the formation and propagation of subsurface cracks. This results in structural damage, such as transverse and vertical cracks, which are especially evident in composite materials [50].

### 4.3. Impact Resistance

In the impact test, the addition of glass fibers (GFs) positively affects energy absorption and enhances the overall resilience. The fibers provide resistance to impact, with a higher fiber content in the impact zone leading to greater resistance. They need to be pulled out and broken (Figure 14c,d and Figure 15c,d), but when a mesh is incorporated into the UPE + 50SiC composite, the samples did not break completely, demonstrating significantly increased resilience. As previously shown in Figure 13, the resilience values indicate that adding SiC and, to a larger extent, GF and mesh, enhances the toughness of UPE. The high resilience corresponds with the increased toughness of the composite.

On the other hand, the PP matrix is inherently tough, so adding 50SiC makes it more rigid, reducing energy absorption and, consequently, resilience (Figure 13). Resilience increases again with the addition of GFs.

### 4.4. Comparison between Composites

Based on the results, composites with a UPE matrix are more suitable than those with a PP matrix for impact resistance, especially when GF is added. The wear resistance of PP matrix composites is higher due to the ductile nature of the PP matrix. This superior wear behavior in PP matrix composites matches that of UPE matrix composites when fibers are added. However, UPE composites have significantly better fire resistance because the UPE matrix carbonizes, whereas the PP matrix melts.

For composites with SiC particles, with or without fiber, the crucial factor is the melting point of the matrix. Therefore, the best material for industrial warehouse coatings is UPE + 50SiC + mesh. This material can be more cost-effective and environmentally friendly if SiC is replaced with materials from the grinding of construction aggregates, which contain a high amount of silicates that are crucial for fire resistance.

## 5. Conclusions

Some general conclusions can be summarized in four points:

(a) Fire resistance:
  - Polypropylene (PP): the fire resistance of PP improves with the addition of SiC and glass fibers (GFs), which form a protective silica layer that insulates and reduces smoke.
  - Polyester resin (UPE): UPE decomposes at high temperatures, but the addition of GF improves its fire resistance by forming protective carbon layers.
(b) Wear behavior:
  - PP matrix composites: Their behavior is derived from the ductile matrix of PP, which results in narrower and shallower wear tracks. This behavior is improved by adding SiC and GF.
  - UPE matrix composites: although the wear of the UPE matrix is relatively high, its wear resistance increases with SiC and GF, forming wedge-shaped agglomerates that reduce the depth of wear.
(c) Impact resistance:
  - Effect of GF: the addition of GF increases energy absorption and resilience.
  - PP versus UPE: PP becomes more rigid with SiC, which reduces resilience, while UPE compounds become more resistant and have increased resilience with SiC and GF, being particularly high when a mesh is added.
(d) The best material for industrial applications:

UPE + 50SiC + mesh: This compound is ideal for industrial warehouse linings due to its superior resistance to fire, wear, and impact. It may be more profitable and ecological to replace SiC with aggregate construction materials that contain silicates.

## Figures and Tables

**Table 1:** Nomenclature of the tested samples.

Matrix	Reinforcement	Nomenclature
PP	50 wt% SiC	PP + 50SiC
	50 wt% SiC + 1 wt% GF	PP + 50SiC + 1GF
	50 wt% SiC + 3 wt% GF	PP + 50SiC + 3GF
UPE	50 wt% SiC	UPE + 50SiC
	50 wt% SiC + 1 wt% GF	UPE + 50SiC + 1GF
	50 wt% SiC + 3 wt% GF	UPE + 50SiC + 3GF
	50 wt% SiC + mesh GF	UPE + 50SiC + mesh

**Table 2:** Time until the flame passed through the PP composites.

PP Composites	Time (s)
PP + SiC	65
PP + 50SiC + 1GF	74
PP + 50SiC + 3GF	105

**Table 3:** Time until the flame passed through the UPE composites.

UPE Composites	Time until the Flame Passed through the Material(s)
UPE + SiC	76
UPE + 50SiC + 1GF	101
UPE + 50SiC + 3GF	120
UPE + 50SiC + mesh	430

**Table 4:** DSC data for PP and its composites.

PP Composites	T_m_ ± 2 (°C)	ΔH_m_ ± 1 (J/g)	Crystallinity (%)
PP	175	63	31
PP + 50SiC	169	38	37
PP + 50SiC + 1GF	169	30	30
PP + 50SiC + 3GF	160	29	30

## Data Availability

Data are contained within the article. The Excel files used in this work and the micrographs produced are available for consultation by contacting the corresponding author.
